# Tricetin Induces Apoptosis of Human Leukemic HL-60 Cells through a Reactive Oxygen Species-Mediated c-Jun N-Terminal Kinase Activation Pathway

**DOI:** 10.3390/ijms18081667

**Published:** 2017-07-31

**Authors:** Ming-Hsien Chien, Jyh-Ming Chow, Wei-Jiunn Lee, Hui-Yu Chen, Peng Tan, Yu-Ching Wen, Yung-Wei Lin, Pei-Ching Hsiao, Shun-Fa Yang

**Affiliations:** 1 Graduate Institute of Clinical Medicine, College of Medicine, Taipei Medical University, Taipei 110, Taiwan; mhchien1976@gmail.com (M.-H.C.); cancercute@yahoo.com.tw (P.T.); highwei168@gmail.com (Y.-W.L.); 2 Department of Medical Education and Research, Wan Fang Hospital, Taipei Medical University, Taipei 116, Taiwan; lwj5905@gmail.com; 3 Division of Hematology and Medical Oncology, Department of Internal Medicine, Wan Fang Hospital, Taipei Medical University, Taipei 116, Taiwan; chow0803@yahoo.com.tw; 4 Department of Urology, School of Medicine, Taipei Medical University, Taipei 110, Taiwan; s811007@yahoo.com.tw; 5 Institute of Medicine, Chung Shan Medical University, Taichung 402, Taiwan; albee55@gmail.com; 6 Department of Urology, Wan Fang Hospital, Taipei Medical University, Taipei 116, Taiwan; 7 School of Medicine, Chung Shan Medical University, Taichung 402, Taiwan; 8 Department of Internal Medicine, Chung Shan Medical University Hospital, Taichung 402, Taiwan; 9 Department of Medical Research, Chung Shan Medical University Hospital, Taichung 402, Taiwan

**Keywords:** tricetin, apoptosis, c-Jun N-terminal kinase, reactive oxygen species, acute myeloid leukemia

## Abstract

Tricetin is a dietary flavonoid with cytostatic properties and antimetastatic activities in various solid tumors. The anticancer effect of tricetin in nonsolid tumors remains unclear. Herein, the molecular mechanisms by which tricetin exerts its anticancer effects on acute myeloid leukemia (AML) cells were investigated. Results showed that tricetin inhibited cell viability in various types of AML cell lines. Tricetin induced morphological features of apoptosis such as chromatin condensation and phosphatidylserine (PS) externalization, and significantly activated proapoptotic signaling including caspase-8, -9, and -3 activation and poly(ADP-ribose) polymerase (PARP) cleavage in HL-60 AML cells. Of note, tricetin-induced cell growth inhibition was dramatically reversed by a pan caspase and caspase-8- and -9-specific inhibitors, suggesting that this compound mainly acts through a caspase-dependent pathway. Moreover, treatment of HL-60 cells with tricetin induced sustained activation of extracellular signal-regulated kinase (ERK) and c-Jun N-terminal kinase (JNK), and inhibition of ERK and JNK by their specific inhibitors respectively promoted and abolished tricetin-induced cell apoptosis. Dichlorofluorescein (DCF) staining showed that intracellular reactive oxygen species (ROS) levels were higher in tricetin-treated HL-60 cells compared to the control group. Moreover, an ROS scavenger, *N*-acetylcysteine (NAC), reversed tricetin-induced JNK activation and subsequent cell apoptosis. In conclusion, our results indicated that tricetin induced cell death of leukemic HL-60 cells through induction of intracellular oxidative stress following activation of a JNK-mediated apoptosis pathway. A combination of tricetin and an ERK inhibitor may be a better strategy to enhance the anticancer activities of tricetin in AML.

## 1. Introduction

Acute myeloid leukemia (AML) is a heterogeneous clonal disorder of hematopoietic progenitor cells and the most common type of leukemia in adults. Despite significant improvements being made in the past three decades in the understanding of leukemia pathogenesis, prognostic factors, drug treatment, and patient care procedures, the prognosis of AML is still not good enough, and estimated 5-year overall survival after a standard chemotherapy is only 38% [1,2]. Chemotherapy resistance and relapse still remain major challenges in treating AML [3]. Thus, a better understanding of the molecular biology of AML is needed, and alternative treatments for AML patients still need to be discovered.

Nowadays, some plant-derived natural products have been used as alternative treatments for leukemias including AML because of their extensive biological activities and comparatively low toxicities [4,5]. Tricetin (5,7,3′,4′,5′-pentahydroxyflavone), a dietary flavonoid in Myrtaceae pollen and *Eucalyptus* honey, appears to have potent anti-inflammatory properties which may be responsible for its beneficial effects [6]. Recently, tricetin has garnered much attention in relation to its anticancer activities such as antiproliferative and antimetastatic activities in many solid tumor cell models including breast [7], liver [8], lung [9], bone [10], and brain [11] tumors. Although it is quite clear that tricetin can inhibit the growth or metastasis of various solid tumor cells, the precise impact of tricetin on nonsolid tumors is still unclear.

Apoptosis is an active process of endogenous programmed cell death. The identified characteristics of apoptosis include morphologic changes such as condensation and fragmentation of nuclei, cell membrane shrinkage, and loosening of organelle positions in the cytoplasm. In addition to morphological changes, sophisticated molecular procedures and mechanisms are also involved. Apoptosis can be initiated either through a death receptor followed by caspase-8 and -10 activation or the mitochondrial pathway involving caspase-9 [12]. One of the hallmarks of cancer is the deregulation of apoptosis; thus increasing apoptosis in tumors is one of the best ways for anticancer agents to treat all types of cancer. Actually, there are several plant-derived anticancer agents such as *Vinca* alkaloids, taxines, and podophyllotoxin already in clinical use [13].

The mitogen-activated protein kinase (MAPK) pathway is an important route that communicates extracellular signals in intracellular responses and was correlated with many physiological processes such as cell growth, differentiation, and apoptosis. In mammalian cells, there are three well-characterized subfamilies of MAPKs: extracellular signal-regulated kinases (ERKs), c-Jun N-terminal kinases (JNKs), and p38 MAPKs [14]. JNK was reported to be phosphorylated/activated after exposure of cells to stressful stimuli, such as irradiation and cancer chemotherapeutics, and it plays an important role in chemotherapeutic drug-mediated apoptosis [15]. Recently, it was reported that a JNK-activation defect confers chemoresistance in solid tumors such as ovarian and liver cancers [16,17]. Notably, involvement of the JNK-activation defect in anthracycline-containing chemotherapy resistance was also characterized in AML, and JNK targeting might be a new therapeutic approach for AML [18].

Although it is entirely clear about the anti-metastatic and anti-tumor growth effects of tricetin in various solid tumor cells, the exact impact of tricetin on nonsolid tumors is still unknown. This is the first study to determine the cell growth-inhibitory activity and molecular mechanisms of tricetin in different French-American-British (FAB) types of AML cells (THP-1, U937, HL-60, and MV4-11). Our results demonstrated that tricetin suppressed proliferation of these four AML cell lines. We found that superoxide was overproduced in HL-60 AML cells during tricetin treatment, which initiated a signal leading to activation of JNK-mediated apoptosis. Moreover, a combination of tricetin and an ERK inhibitor may be a better strategy than tricetin alone for treating AML. This study should provide a scientific basis for the clinical use of tricetin to effectively inhibit AML.

## 2. Results

### 2.1. Tricetin Inhibited Proliferation of Human Acute Myeloid Leukemia (AML) Cells

**Figure 1:**
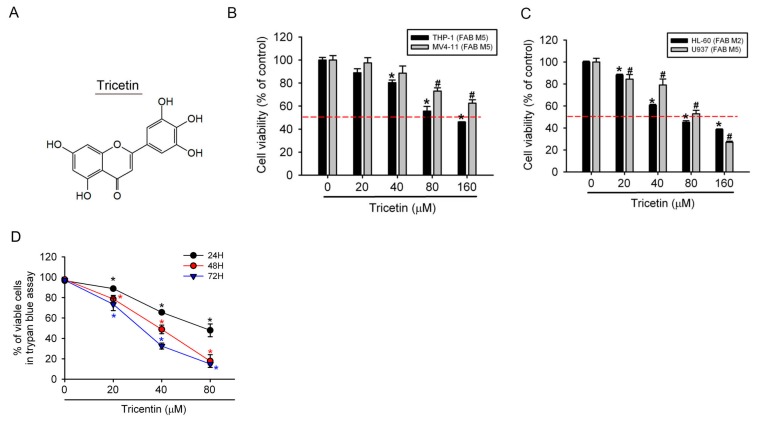
Tricetin treatment results in reduced cell viability of human acute myeloid leukemia (AML) cell lines. (**A**) The chemical structure of tricetin; (**B**,**C**) Four human AML cell lines (HL-60, U937, THP-1, and MV4-11) were treated with the vehicle (DMSO) or tricetin (0~160 μM) in serum-containing medium for 24 h; (**D**) HL-60 cells were treated with different concentrations of tricetin (0~80 μM) for 24, 48, and 72 h. Cell viability was determined by a trypan blue exclusion assay. Results are expressed as multiples of cell viability. Values represent the mean ± standard error (SE) of three independent experiments. *, ^#^ *p* < 0.05, compared to the vehicle groups. The dashed line indicates 50% growth inhibition of cell viability.

The chemical structure of tricetin is shown in Figure 1A. In this study, we first examined the effect of tricetin on the growth of human AML cell lines using the cell counting kit-8 (CCK-8) assay. After treating cells with tricetin for 24 h, the tricetin concentration dependently inhibited the proliferation of four AML cell lines which represent different FAB types (M2: HL-60 and M5: MV4-11, U937, and THP-1) (Figure 1B,C). Among these four AML cell lines, HL-60 cells were the most sensitive to tricetin treatment. Therefore, we chose HL-60 cells for subsequent experiments. We further studied the long-term antiproliferative potential of tricetin against HL-60 cells by trypan blue exclusion assay. As illustrated in Figure 1D, tricetin time- and concentration-dependently suppressed the growth of cultured HL-60 cells.

### 2.2. Tricetin Treatment Results in the Apoptosis of HL-60 AML Cells

**Figure 2:**
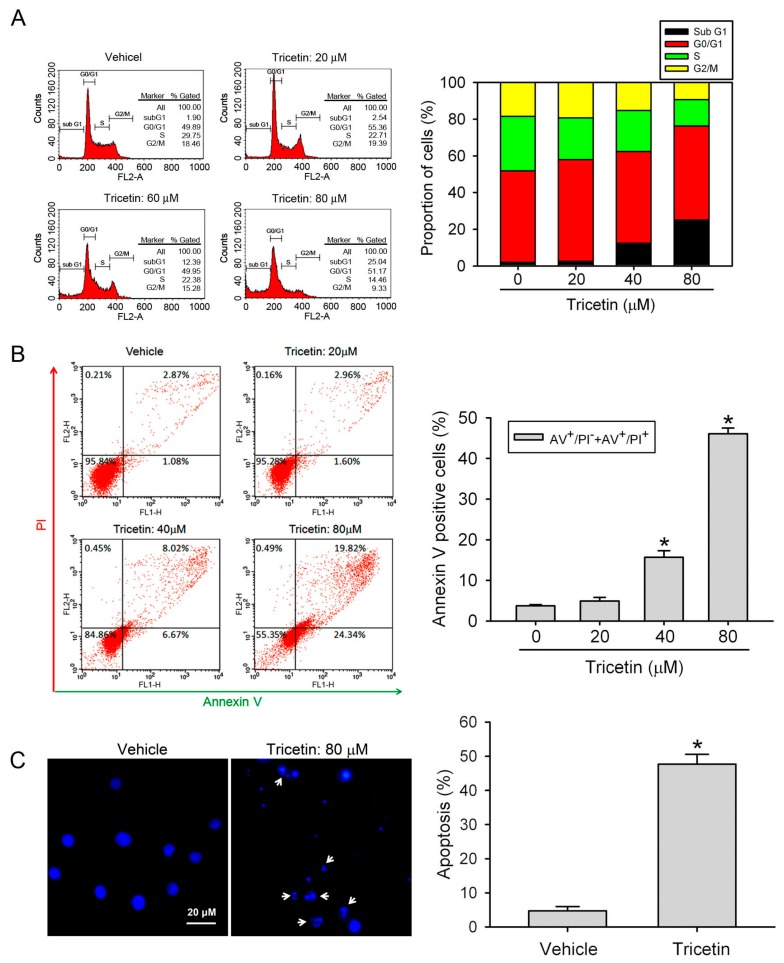
Effect of tricetin on HL-60 cell-cycle regulation and apoptosis. (**A**) HL-60 cells were treated with different concentrations of tricetin (0~80 μM) for 24 h. The cell-cycle phase distribution and cell death in the sub-G_1_ phase were analyzed by fluorescence-activated cell sorting (FACS) after propidium iodide (PI) staining. Data are shown as the cell-cycle distribution profile by FACS and the percentage distribution of cells in the sub-G_1_, G_0_/G_1_, S, and G_2_/M phases; (**B**) HL-60 cells were treated with different concentrations of tricetin (0~80 μM) for 24 h. Quantitative analysis of cell apoptosis by FACS after staining with Annexin-V and PI. In the dot plots, percentages of Annexin-V^+^/PI^−^ (cells in early apoptosis, bottom right quadrant) and Annexin-V^+^/PI^+^ (cells in late apoptosis, top right quadrant) are shown (**C**) HL-60 cells were treated with 80 μM tricetin for 24 h and analyzed by fluorescence microscopy after 4′,6-diamidino-2-phenylindole (DAPI) staining. White arrows indicate apoptotic HL-60 cells. Percentage of apoptotic cells expresses ratio of apoptotic cells to the total cell number. For each sample 200 cells were assessed. Data are expressed as the mean ± SE of three independent experiments. * *p* < 0.05, compared to the vehicle group.

Physiological cell death is characterized by an apoptotic morphology, including cell shrinkage, nuclear condensation and fragmentation, translocation of phosphatidylserine (PS) to the extracellular membrane, dynamic membrane blebbing, and loss of adhesion to neighbors or to the extracellular matrix [19]. To determine whether tricetin-induced growth inhibition resulted from cell apoptosis, HL-60 cells were treated with tricetin (0~80 μM) for 24 h, and we found that tricetin induced concentration-dependent increases of the sub-G_1_ population (Figure 2A). Moreover, we performed Annexin-V/PI double staining to identify translocation of PS in HL-60 cells. As shown in Figure 2B, early (PI-negative/Annexin-V-positive) and late apoptotic cells (PI-positive/Annexin-V-positive) all dramatically increased after respectively treating HL-60 cells with 40 and 80 μM tricetin. Because the tricetin-induced increase in the PI-positive/Annexin-V-positive population also might have been due to induction of necrosis, we next examined the effect of tricetin on cell morphology by using fluorescence microscopy. Treatment of cells with 80 μM tricetin for 24 h showed morphologies characteristic of apoptosis including chromatin condensation and apoptotic bodies formation (Figure 2C, arrows). These results are all hallmarks of cell apoptosis and confirmed that tricetin can induce apoptotic cell death in HL-60 cells.

### 2.3. Tricetin Induces Caspase-Dependent Apoptotic Cell Death in HL-60 Cells

**Figure 3:**
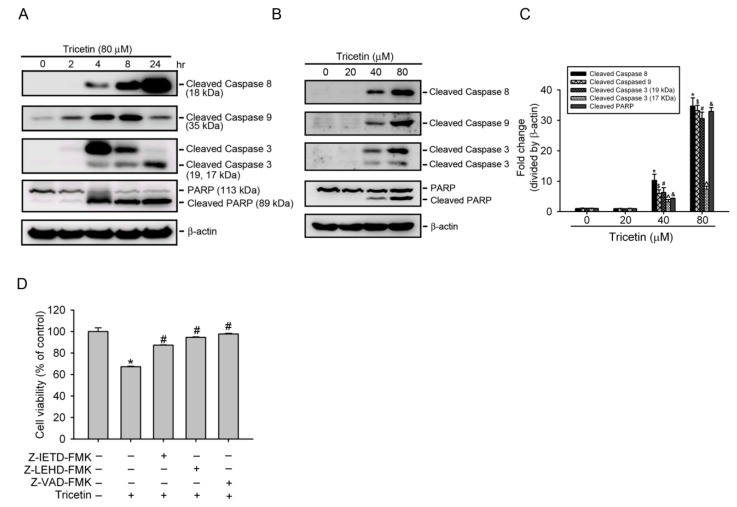
Tricetin induces caspase-dependent apoptotic cell death in HL-60 cells. (**A**) Expression levels of cleaved caspases-3, -8, and -9, and poly(ADP-ribose) polymerase (PARP) were assessed by a Western blot analysis after treatment with 80 μM tricetin for indicated time points; (**B**) Activated caspase-8, -9, and -3, and cleaved PARP protein expressions were upregulated in a concentration-dependent fashion after treatment of HL-60 cells with various concentrations of tricetin (0~80 μM) for 8 h; (**C**) Quantitative results of cleaved caspase-3, -8, and -9, and PARP protein levels, which were adjusted to the β-actin protein level and expressed as multiples of induction beyond each respective control. Values are presented as the mean ± SE of three independent experiments. *, ^#^, ^&^, ^^^, ^$^ *p* < 0.05, compared to the vehicle control groups; (**D**) Cells were treated with 40 μM tricetin for 24 h in the presence or absence of 50 μM Z-VAD-FMK, Z-LEHD-FMK, or Z-IETD-FMK. Cell proliferation was determined by an MTS assay. Data are presented as the mean ± SE of three independent experiments performed in triplicate. * *p* < 0.05, control vs. tricetin; ^#^ *p* < 0.05, tricetin vs. Z-VAD-FMK, Z-LEHD-FMK, or Z-IETD-FMK plus tricetin.

The apoptotic procedure is carried out by a group of the highly conserved caspases, and modulation of the mechanisms of caspase activation and suppression is a key molecular target in chemoprevention, since these procedures lead to apoptosis [20]. To clarify the mechanisms underlying tricetin-induced apoptosis in AML cells, activation of initiator (caspases-9 and -8) and executioner (caspase-3) caspases was detected. Figure 3A shows that exposure of HL-60 cells to tricetin (80 μM) for indicated time points caused hydrolysis of procaspases-8, -9, and -3 and cleavage of caspase-3’s substrate, PARP, in time-dependent manners. Moreover, treatment of HL-60 cells with different concentrations (0~80 μM) of tricetin for 8 h also resulted in concentration-dependent increases in activated caspases-8, -9, and -3, and cleaved PARP (Figure 3B,C). We next further investigated whether activation of caspases is necessary for tricetin-induced apoptosis of HL-60 cells by an MTS assay using specific inhibitors that respectively inhibit caspase-9 (Z-LEHD-FMK), caspase-8 (Z-IETD-FMK), or a broad-spectrum caspase (Z-VAD-FMK). As shown in Figure 3D, all caspase inhibitors significantly attenuated 40 μM tricetin-induced inhibition of proliferation. Taken together, the results indicated that tricetin induced rapid and time- and concentration-dependent apoptosis in a caspase-dependent pathway.

### 2.4. Mitogen-Activated Protein Kinases Involved in Tricetin-Regulated Apoptotic Cell Death

**Figure 4:**
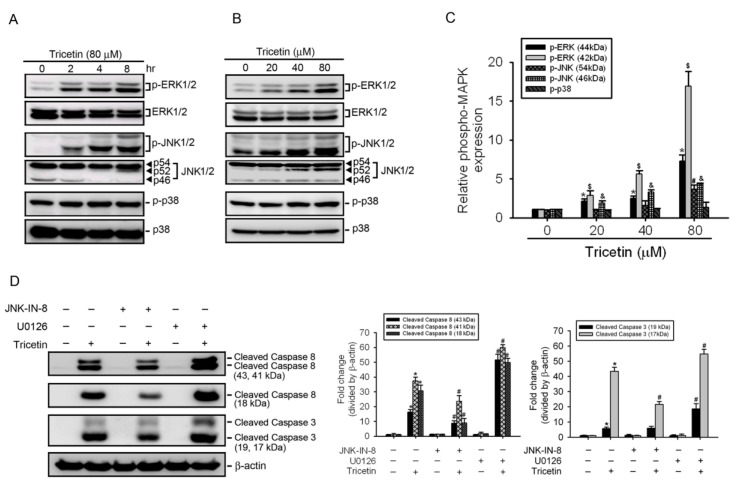

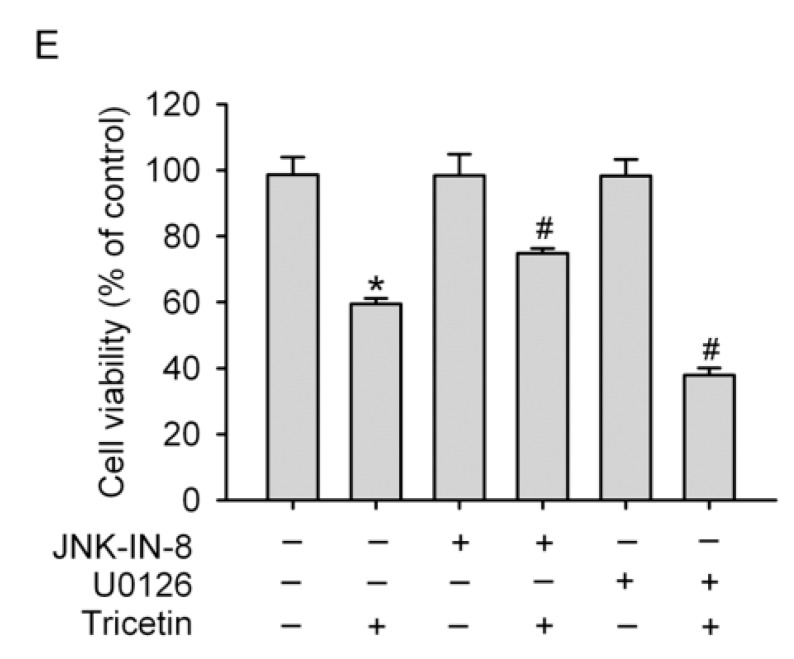
Role of mitogen-activated protein kinases (MAPKs) in tricetin-induced apoptotic cell death in HL-60 cells. (**A**,**B**) Phosphorylation levels of extracellular signal-regulated kinase (ERK)1/2, p38, and c-Jun N-terminal kinase (JNK)1/2 were assessed by a Western blot analysis after treatment of HL-60 cells with 80 μM tricetin for indicated time points (**A**) or with various concentrations of tricetin (0~80 μM) for 8 h (**B**,**C**) Quantitative results of phopho-ERK1/2, p38, and JNK1/2 protein levels, which were adjusted to the total ERK1/2, p38, and JNK1/2 protein levels and expressed as multiples of induction beyond each respective control. Values are presented as the mean ± SE of three independent experiments. *, ^#^, ^&^, ^$^ *p* < 0.05, compared to the vehicle control groups; (**D**,**E**) HL-60 cells were pretreated with or without 5 μM U0126 or 1 μM JNK-IN-8 for 1 h followed by tricetin (40 μM) treatment for an additional 24 h. Expression levels of cleaved caspases-3, -8, and cell viability were respectively determined by a Western blot analysis (**D**, **left** panel) and a CCK-8 assay (**E**). Quantitative results of cleaved caspase-3 and -8 protein levels, which were adjusted to the β-actin protein level and expressed as multiples of induction beyond each respective control (**D**, **right** panel). Values represent the mean ± SE of three independent experiments. * *p* < 0.05, control vs. tricetin; ^#^ *p* < 0.05, tricetin vs. U0126 or JNK-IN-8 plus tricetin.

The MAPK signaling pathway, such as JNK1/2 and/or p38 MAPK, was reported to be participated in the caspase-mediated apoptotic effect induced by different traditional Chinese herbs in various cancer types, including AML [21,22,23]. In contrast, ERK was shown to correlate with the proliferation and drug resistance of hematopoietic cells [24]. Therefore, we determined whether activation of MAPKs was affected in tricetin-treated HL-60 cells, and found that tricetin induced activation of JNK1/2 and ERK1/2, but not p38 MAPK in concentration- and time-dependent manners (Figure 4A–C). Surprisingly, tricetin also induced the formation of cleaved p54 JNK and p52 JNK at 8 h after the addition of 40 or 80 μM tricetin (Figure 4A,B). To further investigate relationships among tricetin-induced activation of caspases and MAPKs, HL-60 cells were pretreated with U0126 (an ERK inhibitor) or JNK-IN-8 (a JNK inhibitor) for 1 h, treated with 40 μM tricetin for another 24 h, and then analyzed by Western blotting. As shown in Figure 4D JNK-IN-8 and U0126 respectively attenuated and increased tricetin-induced caspase-8 and -3 activation. Moreover, we found that JNK-IN-8 and U0126 also respectively attenuated and enhanced tricetin-induced inhibition of proliferation (Figure 4E). These findings suggest that activation of ERK1/2 and JNK1/2 might play opposite roles in tricetin-mediated caspase activation and cell death in HL-60 cells.

### 2.5. Tricetin-Induced Intracellular Oxidative Stress as an Initial Signal for JNK-Mediated Apoptosis in HL-60 Cells

**Figure 5:**
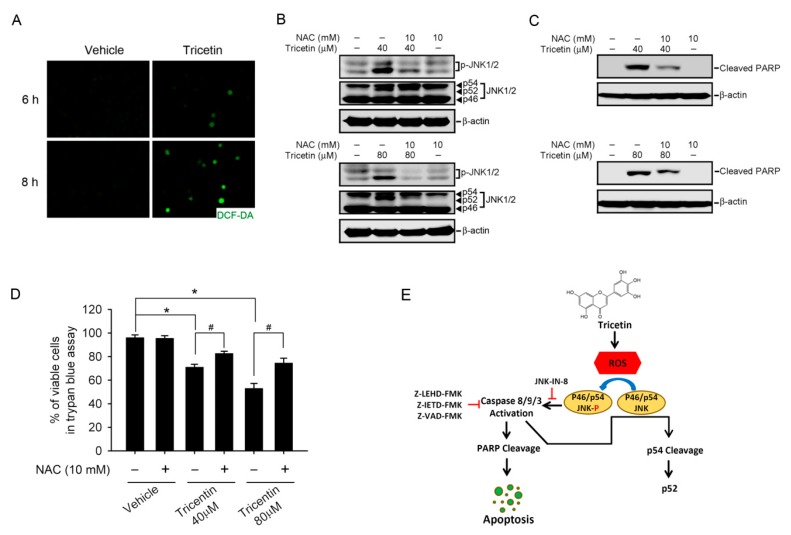
Tricetin-induced intracellular oxidative stress as an initial signal for c-Jun N-terminal kinase (JNK)-mediated apoptosis in HL-60 cells. (**A**) HL-60 cells were treated with 80 μM tricetin for the indicated time, and then the total reactive oxygen species (ROS) level was measured by H_2_DCF-DA staining under a fluorescence microscope. Original magnification, 200×; (**B**,**C**) HL-60 cells were pretreated with or without 10 mM N-acetylcysteine (NAC) for 1 h followed by treatment with 40 or 80 μM tricetin for 8 h; levels of JNK1/2, p-JNK1/2, cleaved poly(ADP ribose) polymerase (PARP), and β-actin were detected by a Western blot analysis; (**D**) HL-60 cells were pretreated with or without 10 mM NAC for 1 h followed by treatment with 40 or 80 μM tricetin for 12 h Trypan blue exclusion assay was used to quantify the cell viability change in each group. Values represent the mean ± SE of three independent experiments. * *p* < 0.05, control vs. tricetin; ^#^ *p* < 0.05, tricetin vs. NAC plus tricetin; (**E**) Proposed signal transduction pathways by which tricetin induces apoptosis of acute myeloid leukemias (AML) cells. The antileukemic activity of tricetin was attributed to its apoptosis induction by increasing ROS production and further inducing activation of JNK and caspases-8, -9, and -3. The p54 JNK cleavage was also induced by tricetin-mediated ROS upregulation. Black arrows indicate induced effects and red t-bar indicate inhibitory effects.

A recent report suggested that JNK activation is associated with ROS-induced apoptosis of chronic myelogenous leukemia cells [25]. In order to test whether tricetin leads to ROS generation in AML cells, HL-60 cells were treated with tricetin for indicated time points, and cellular ROS were monitored with H_2_DCFDA, a fluorescent, redox-sensitive dye. Our results showed that compared to the control group, treatment of cells with 80 μM tricetin dramatically increased dichlorofluorescein (DCF) fluorescence at 6 and 8 h after treatment (Figure 5A). To further dissect the correlation between ROS production and JNK activation in tricetin-mediated cell death, the results showed that pretreating HL-60 cells with the antioxidant, NAC, significantly blocked 40 and 80 μM tricetin-induced JNK activation and cleavage (Figure 5B) and subsequently reversed tricetin-induced cell death as evidenced from decreases in tricetin-induced PARP cleavage (Figure 5C), procaspase-9 and -3 hydrolysis (Figure S1), and cell proliferation inhibition (Figure 5D).

## 3. Discussion

AML is a non-solid tumor with high mortality rates. At present, conventional chemotherapy has favorable outcomes; however, the response is limited by progressive resistance and a number of side effects [2]. There is great interest in therapy with drugs of plant origins, because traditional medicines have less toxicity and fewer side effects [13]. Our current study showed that tricetin, a dietary flavonoid in Myrtaceae pollen and *Eucalyptus* honey, reduced HL-60 leukemic cell growth through increasing endogenous ROS production, which is the initial signal to activate JNK1/2-mediated cell apoptosis. Therefore, tricetin could have physiologic value taken in a natural food form.

The enhancement of ROS production has long been associated with the apoptotic response induced by several anticancer agents [26]. Targeting ROS levels could be a novel approach for AML treatment since ROS levels are higher in malignant cells than in non-malignant cells [27]. Furthermore, increasing oxidative stress is emerging as a promising therapy for leukemia [28]. For example, our previous study showed that quercetin induced cell death of HL-60 cells in vitro and in vivo through induction of intracellular oxidative stress [29]. Moreover, several studies also showed that clinically achievable concentrations (1~2 μM) of a chemotherapeutic drug, arsenic trioxide (As_2_O_3_), induced apoptosis through an ROS-dependent pathway in acute promyelocytic leukemia (APL or AML M3) [30]. Accumulation of intracellular ROS by quercetin or As_2_O_3_ led to dissipation of the mitochondrial membrane potential (MMP), release of cytochrome c from mitochondria, subsequent activation of caspase-9, and ultimately apoptotic cell death [31,32]. Our study showed that tricetin can induce caspase-9-mediated cell death, but the effect of tricetin on the MMP in AML cells will be investigated in our future work. Recently, flavonoids isolated from grape or *Citrus paradisi* Macfadyen were reported to enhance the antileukemic activity of As_2_O_3_ [33,34]. In addition, several mild ROS generators were also reported to facilitate As_2_O_3_-induced apoptosis of different types of cancer cells as well as As_2_O_3_-resistant leukemic cell lines [28]. The therapeutic potential of tricetin combined with As_2_O_3_ in AML treatment will also be further investigated.

Chemotherapeutic resistance and relapse remain major challenges in treating AML. Previous reports indicated that JNK activation is the critical step for the chemotherapy drug, anthracycline, to trigger apoptosis in AML, and a JNK-activation defect confers chemoresistance in AML [18]. Mitochondrial release of ROS upon different apoptotic stimulations can lead to activation of JNK [25,35]. In response to ROS, JNKs induce the phosphorylation and inactivation of antiapoptotic proteins such as B-cell lymphoma 2 (Bcl-2) and B-cell lymphoma-extra large (Bcl-XL) [35]. Previous reports indicated that Bcl-2 and Bcl-XL can antagonize ROS generation and protect cells from ROS-mediated apoptosis [36]. In addition, JNK was also reported to modify the composition of the Bcl-2-associated X (Bax)/Bcl-2 complex through increasing the expression of Bax, leading to formation of Bax homodimers resulting in dissipation of mitochondrial membrane potential [37]. Actually, Hsu et al. have demonstrated that tricetin-induced liver cancer cells apoptosis is associated with Bcl-2 family members-regulated mitochondrial pathway, which is mediated by ROS generation and, subsequently, JNK activation [8]. Our current study demonstrated that tricetin induced leukemic cell death through inducing ROS-mediated JNK activation. The effect of tricetin on Bcl-2 family members and determining whether tricetin can sensitize anthracycline-induced cell death in AML through JNK activity modulation are worthy of evaluating in future work. In addition to JNK activation, we also observed the conversion of p52 from p54 JNK after tricetin treatment in AML cells. This finding is in line with previous reports in MOLT-4 and U937 leukemia cells which showed X-irradiation or heat treatment can induce caspase-3-mediated cleavage of p54 JNK, and this JNK cleaved form still harbors its kinase activity [38].

In addition to JNK, our study also showed that tricetin can induce ROS-mediated ERK activation in HL-60 cells (Figure S2). ERK activation was reported to be a key pathway that protects cancer cells from apoptosis including in leukemia [39]. Previous reports indicated that ERK1/2 can promote leukemic cell survival by enhancing the activity of antiapoptotic proteins including Bcl-2, Bcl-XL, Mcl-1, inhibitor of apoptosis protein (IAP), and repressing the activity or expression of proapoptotic proteins, such as Bad, Bim, and caspase-9 [39,40]. Recently, evidence has also shown that ERK1/2 can have proapoptotic functions in response to different stimuli such as etoposide, bufalin, shikonin, doxorubicin, and apigenin in different cancer types [40]. Although our present results indicated that tricetin (40 and 80 μM) can induce apoptosis of HL-60 cells, results showed that ERK can also be activated instead of inhibited by higher concentrations (40 and 80 μM) of tricetin. Moreover, inhibition of the ERK pathway by a specific inhibitor further enhanced the apoptosis-inducing effect of tricetin in AML cells. Consistent results from previous studies also indicated that an ERK inhibitor enhances docetaxel- and DAPT-induced apoptosis in androgen-independent prostate cancer and gastric cancer, respectively [41,42]. Taken together, we propose that ERK activation induced by higher concentrations of tricetin may have been due to a cell-derived protective effect against the toxic effects of tricetin. According to these findings, we suggest that a combination of tricetin and an ERK inhibitor may be a good strategy for preventing the growth of AML cells.

In addition to MAPKs, a breast cancer resistance protein (BCRP/ABCG2) was reported to be overexpressed by stem cells in leukemias, potentially contributing to their resistance to eradication by chemotherapy or targeted therapies and to be associated with a poor response to conventional chemotherapy and increased risk of relapse [43]. Recently, tricetin was identified as a novel flavonoid ABCG2 inhibitor [44], but the effect of tricetin on the stemness of leukemia should be further investigated.

## 4. Materials and Methods

### 4.1. Materials

Tricetin was purchased from Extrasynthese (Genay, France). An 80-mM stock solution of tricetin was made in dimethyl sulfoxide (DMSO) (Sigma-Aldrich, St. Louis, MO, USA) and stored at −20 °C. The final concentration of DMSO for all treatments was <0.5%. 2′,7′-Dichlorofluorescein diacetate (DCFDA), N-acetylcysteine (NAC), z-IETD-FMK (a caspase-8 inhibitor), zLEHD-FMK (a caspase-9 inhibitor), and a general inhibitor of caspases (zVAD-FMK) were purchased from Sigma-Aldrich. Antibodies against poly(ADP-ribose) polymerase (PARP), phosphorylated (p)-c-Jun N-terminal kinase (JNK), JNK1/2, p-extracellular signal-regulated kinase (ERK)1/2, caspase-9, caspase-3, and caspase-8 were purchased from Cell Signaling Technology (Beverly, MA, USA). Anti-p-p38, p38, and β-actin antibodies were obtained from BD Biosciences (San Jose, CA, USA).

### 4.2. Cell Culture

The human MV4-11, HL-60, U937, and THP-1 AML cell lines were purchased from American Type Culture Collection (Manassas, VA, USA). All cell lines were cultured in RPMI 1640 medium supplemented with 10% heat-inactivated fetal bovine serum (FBS; Gibco, Grand Island, NY, USA), 0.1 mM nonessential amino acids, 2 mM l-glutamine, 100 U/mL penicillin, and 100 μg/mL streptomycin.

### 4.3. In Vitro Cytotoxicity Assay

Four AML cells (MV4-11, HL-60, U937, and THP-1) were cultured in 96-well plates containing complete media and treated with different concentrations of tricetin (0, 20, 40, 80, and 160 μM) for 24 h, and cell viabilities were examined using a Cell Counting Kit-8 (CCK-8) (Sigma-Aldrich) or MTS (Promega, Madison, WI, USA) assay. The absorbance (A) was read at 450 nm (CCK-8 assay) or 490 nm (MTS assay) using an enzyme-linked immunosorbent assay (ELISA) reader (MQX200; Bio-Tek Instruments, Winooski, VT, USA). The cell viability rate (multiple) was analyzed by the formula: A_450 or 490, tricetin_/A_450 or 490, vehicle_.

### 4.4. In Vitro Cell Viability Assay

The effect of tricetin on the viability of HL-60 cells was determined by a trypan blue dye exclusion assay. Briefly, the HL-60 cells were plated at a density of 5 × 10^3^ in 96-well plates containing complete media and treated with tricetin, or tricetin combined NAC. After incubation for indicated time points, cells were collected and an aliquot of cell suspension was mixed with an equal volume of trypan blue and cells were counted under the microscope.

### 4.5. Flow Cytometric Analysis of DNA Contents

HL-60 cells (2 × 10^6^/mL) were treated with vehicle or tricetin (0, 20, 40, and 80 μM) for 24 h and then cells were collected and fixed by 70% ethanol. Next, cells were incubated with propidium iodide (PI) buffer (4 μg/mL PI, 0.5 mg/mL RNase A, and 1% Triton X-100 in phosphate-buffered saline (PBS)) for 30 min at 37 °C in the dark followed by filtration through a 40-μm nylon filter (Falcon, San Jose, CA, USA). The cell cycle distribution was analyzed for 10^4^ collected cells by a FACS Vantage flow cytometer that uses the Cellquest acquisition and analysis program (Becton-Dickinson FACS Calibur, San Jose, CA, USA). The proportion of nuclei in each phase of the cell cycle was determined, and apoptotic cells with hypodiploid DNA peak were catched in the sub-G_1_ region.

### 4.6. 4,6-Diamidino-2-phenylindole (DAPI) Staining

Morphological changes in the nuclear chromatin of cells undergoing apoptosis were visualized following DNA staining using the fluorescent dye 4,6-diamidino-2-phenylindole (DAPI, Sigma). After incubation for 24 h with tricetin, HL-60 cells were fixed with methanol, stained with the DAPI solution for 10 min at room temperature, and examined under a fluorescence microscope. Apoptotic cells exhibited morphological features of apoptosis including nuclear fragmentation and chromatin condensation. The percentage of apoptotic cells were calculated as the ratio of apoptotic cells to total cells counted.

### 4.7. Apoptosis Assays

Apoptotic cell death was determined by Annexin-V-fluorescein isothiocyanate (FITC)/PI double staining, using an FITC-labeled Annexin-V/PI Apoptosis Detection kit (BD Biosciences, San Jose, CA, USA) following the manufacturer’s guidelines. HL-60 cells were exposed to tricetin at the indicated concentration for 24 h, cells were then washed with PBS following trypsinization, resuspended in 100 μL of binding buffer (10 mM HEPES/NaOH, 140 mM NaCl, and 2.5 mM CaCl_2_ at pH 7.4) and stained with 5 μL of FITC-conjugated Annexin-V and 5 μL of PI (50 μg/mL) for 30 min at room temperature, and then 400 μL of binding buffer was added. Apoptotic cells were analyzed by flow cytometry with a FACScan system flow cytometric analysis. Data acquisition and analysis were performed in a Becton-Dickinson FACSCalibur flow cytometer using CellQuest software (BD Biosciences).

### 4.8. Preparation of Total Cell Extracts and Western Blot Analysis

Cell lysates were prepared as previously described. The protein content was determined with the Bio-Rad protein assay reagent using bovine serum albumin as a standard. Equal amounts of protein extracts (10~50 μg) were boiled in Laemmli sample buffer, separated on sodium dodecylsulfate (SDS) polyacrylamide gels, electrophoretically transferred to polyvinylidene fluoride membranes (Millipore, Belford, MA, USA) and incubated with the indicated primary antibodies at 4 °C overnight. Blots were then further incubated with a horseradish peroxidase (HRP)-conjugated secondary antibodies for 1 h at room temperature. Signals were detected through enhanced chemiluminescence (ECL) Western blotting detection reagents (Millipore, Billerica, MA, USA).

### 4.9. Measurement of ROS Production

HL-60 cells were treated with tricetin (80 μM) for 6 and 8 h; then ROS production was detected by staining with an ROS probe (5 μM DCFDA) in RPMI 1640 medium for 30 min. Cells were then washed with PBS or medium and ROS production of DCFDA-preloaded cells were captured with a fluorescence microscope (Nikon Eclipse TE 300, Tokyo, Japan).

### 4.10. Statistical Analysis

Data points represent the mean ± standard error (SE). We performed statistical analyses with Student *t* test was used to compare data between two groups. *p* values of <0.05 were considered statistically significant.

## 5. Conclusions

In conclusion, we first report that tricetin possesses an antileukemic effect on AML cells, and that phenomenon stems from induction of intracellular oxidative stress, which initiates a signal leading to activation of JNK and induces caspase-dependent apoptosis; the mechanism is schematically illustrated in Figure 5E. In addition, we also found that a combination of tricetin and an ERK inhibitor presented a better apoptosis-inducing effect on AML cells than tricetin treatment alone. Our discovery of this novel mechanism of tricetin not only gives further insights into its anticancer potential against hematological malignancies, but also contributes to developing tricetin as a chemopreventive or chemotherapeutic agent in managing human AML.

## Supplementary Materials

Supplementary materials can be found at www.mdpi.com/1422-0067/18/8/1667/s1.

## Abbreviations

AML	Acute myeloid leukemia
DCF	Dichlorofluorescein
DMSO	Dimethyl sulfoxide
ERK	Extracellular signal-regulated kinase
JNK	c-Jun N-terminal kinase
NAC	*N*-Acetylcysteine
PARP	Poly(ADP-ribose) polymerase
PI	Propidium iodide
PS	Phosphatidylserine
ROS	Reactive oxygen species
