# Supplementary material for: Tricetin Induces Apoptosis of Human Leukemic HL-60 Cells through a Reactive Oxygen Species-Mediated c-Jun N-Terminal Kinase Activation Pathway
Source: Int J Mol Sci. 2017 Jul 31;18(8):1667. doi: 10.3390/ijms18081667 (PMC5578057; doi:10.3390/ijms18081667)
Supplement: Supplementary file 1 [file ijms-18-01667-s001.pdf]

Supplemental Information

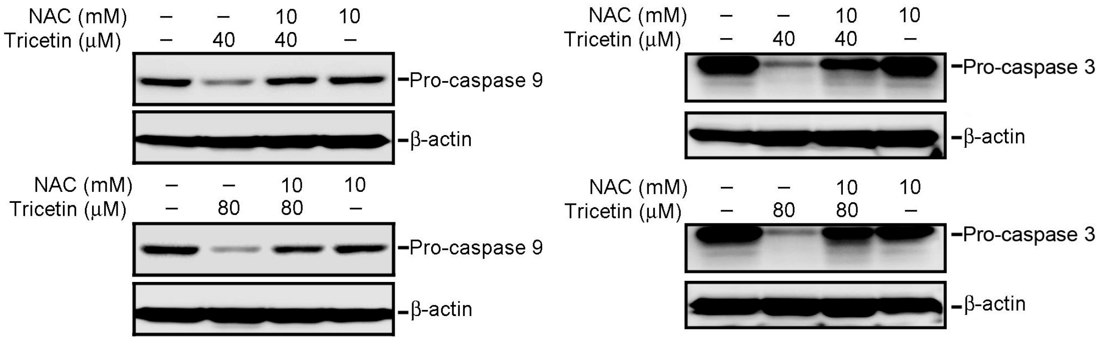

Figure S1. Tricetin induces reactive oxygen species (ROS)-mediated procaspase-9 and -3 hydrolysis in HL-60 cells. HL-60 cells were pretreated with or without 10 mM N-acetylcysteine (NAC) for 1 h followed by treatment with 40 or 80 μM tricetin for 8 h; levels of procaspases-9 and -3, and β-actin were detected by a Western blot analysis.

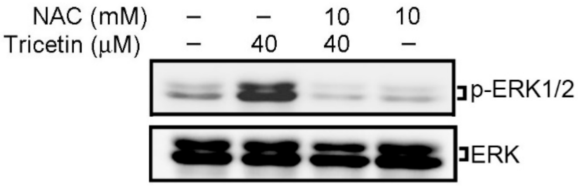

Figure S2. Tricetin induces reactive oxygen species (ROS)-mediated ERK1/2 activation in HL-60 cells. HL-60 cells were pretreated with or without 10 mM N-acetylcysteine (NAC) for 1 h followed by treatment with 40 μM tricetin for 8 h; levels of phopho-ERK1/2, and ERK1/2 were detected by a Western blot analysis.
